# Supplementary figures and images for: Joint effects of genes underlying a temperature specialization tradeoff in yeast
Source: PLoS Genet. 2021 Sep 14;17(9):e1009793. doi: 10.1371/journal.pgen.1009793 (PMC8462698; doi:10.1371/journal.pgen.1009793)

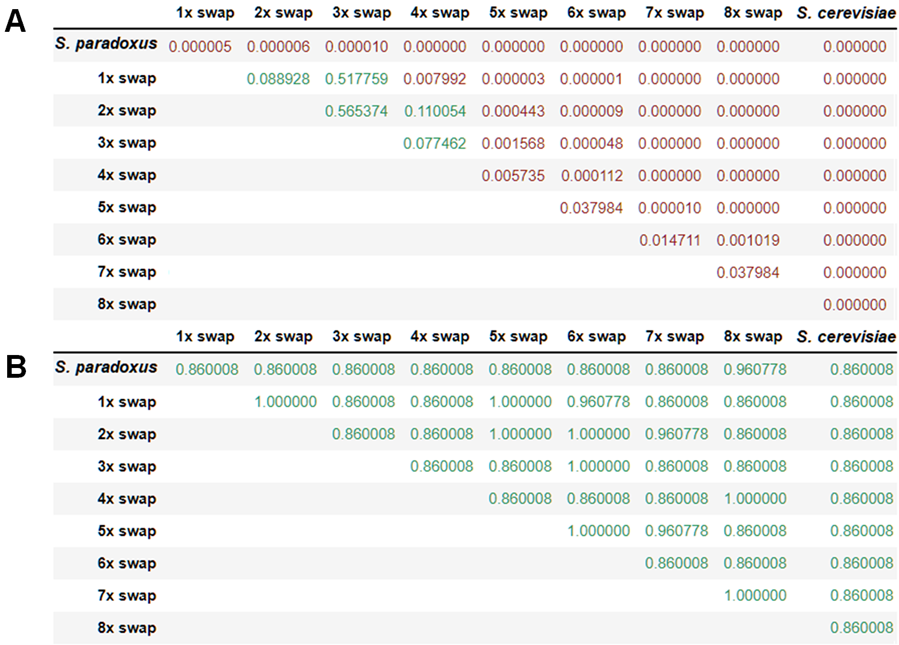

Supplement: S1 Table — (A) Each cell reports the results of a one-sided Wilcoxon test comparing growth efficiency at 39°C between the indicated strains, in Fig 1 of the main text. Multiple testing was corrected for using the Benjamini-Hochberg method. (B) Data are as in A except that analysis was of growth efficiency at 28°C from S1 Fig, and two-sided Wilcoxon tests were applied. (TIF) [file pgen.1009793.s001.tif]

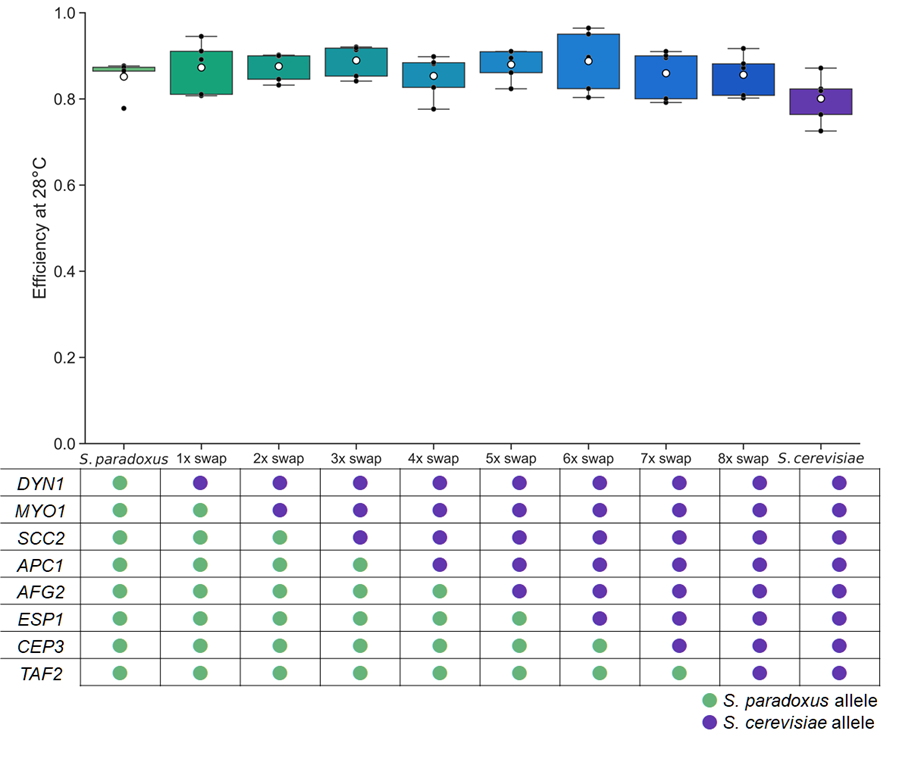

Supplement: S1 Fig — Data and symbols are as in Fig 1 of the main text except that growth was measured at 28°C. Statistical analyses are reported in S1B Table. (TIF) [file pgen.1009793.s004.tif]

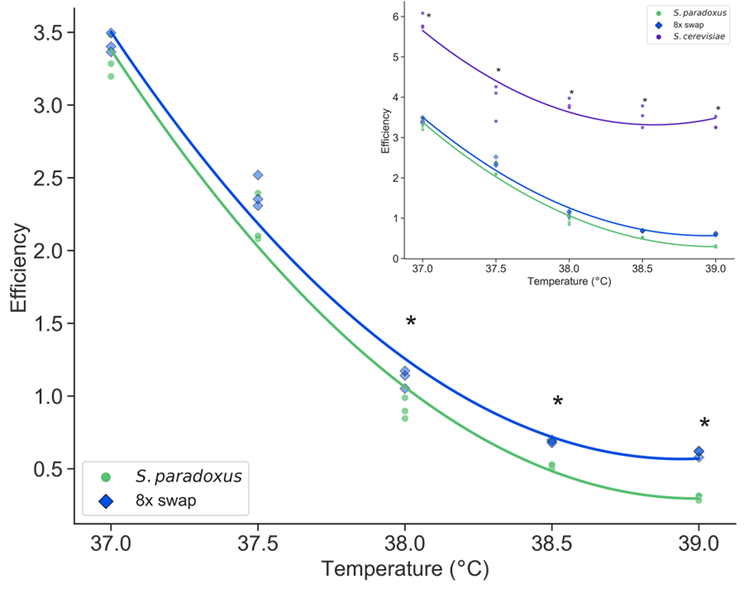

Supplement: S2 Fig — In a given panel, each trace reports growth efficiency, the cell density after a 24-hour incubation at the indicated temperature as a difference from the starting density, of the wild-type of the indicated species or the S. paradoxus strain harboring eight thermotolerance loci from S. cerevisiae (8x swap). Lines are the result of a polynomial regression on the points (S2 Table). *, Wilcoxon p ≤ 0.0404. (TIF) [file pgen.1009793.s005.tif]

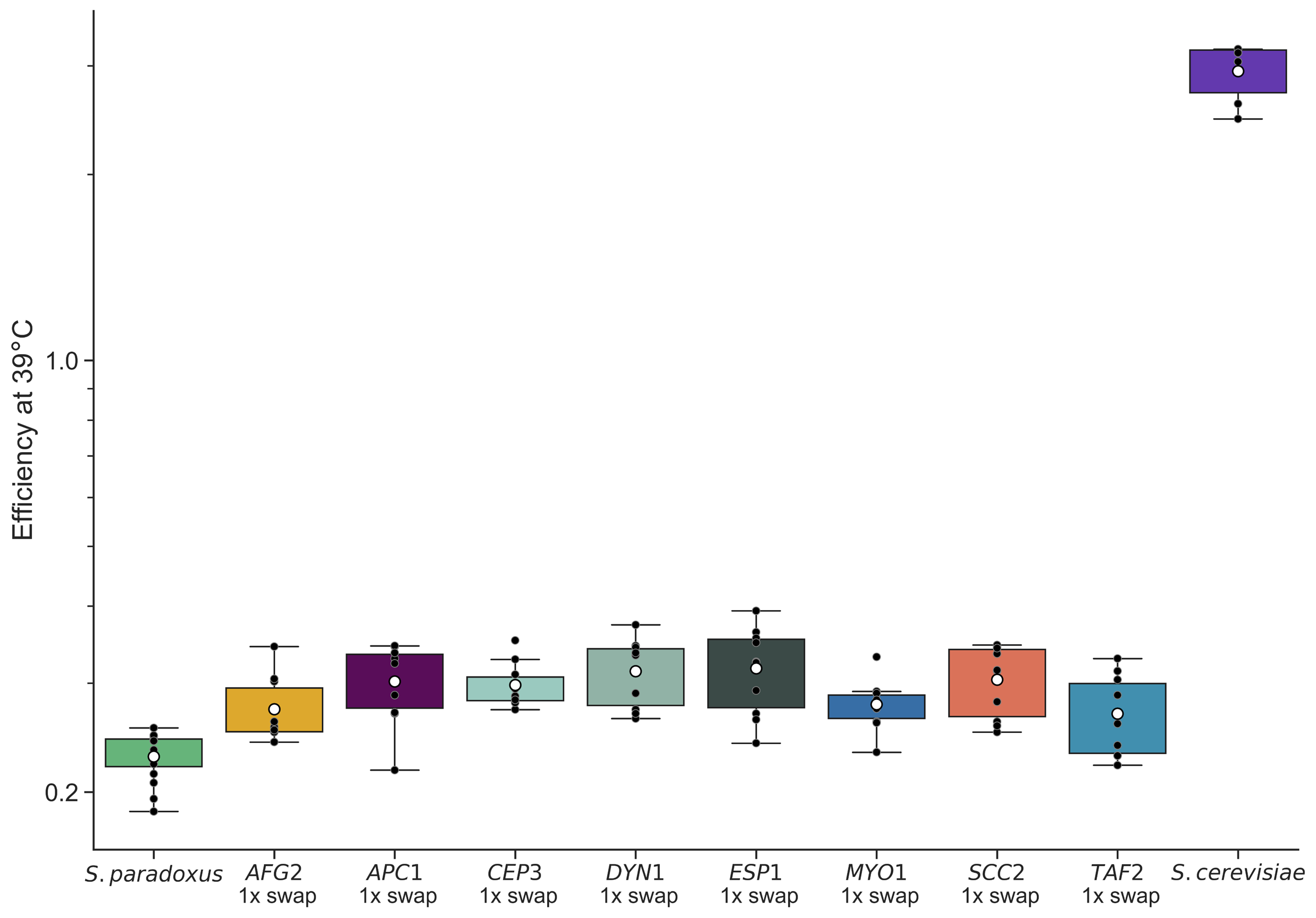

Supplement: S3 Fig — Data and symbols are as in the main panel of Fig 1 except that each column reports results from the indicated wild-type strain or a strain of S. paradoxus harboring the S. cerevisiae allele of the indicated single gene and the y-axis is log-scaled. All comparisons to S. paradoxus had one-sided Wilcoxon p < 0.01 after correction for multiple testing. (TIF) [file pgen.1009793.s006.tif]

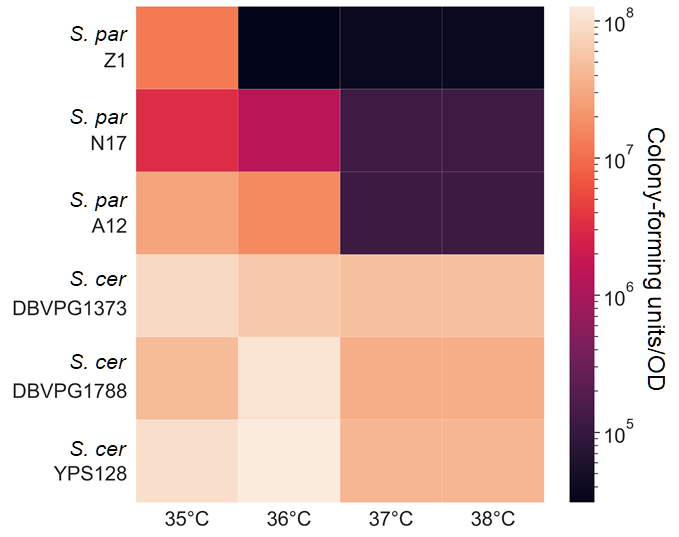

Supplement: S4 Fig — Each cell reports viability after heat treatment of the indicated strain and species: the number of colonies formed on solid medium from 1 mL of heat-treated liquid culture in logarithmic growth, normalized by the turbidity of the latter. S. paradoxus Z1, N17, and A12 are isolates from UK, Russia, and Quebec, respectively; S. cerevisiae strains DBVPG1373, DBVPG1788, YPS128 are from the Netherlands, Finland, and Pennsylvania, respectively. Viability was different between species at Wilcoxon p < 0.00002 for all temperatures. (TIF) [file pgen.1009793.s007.tif]

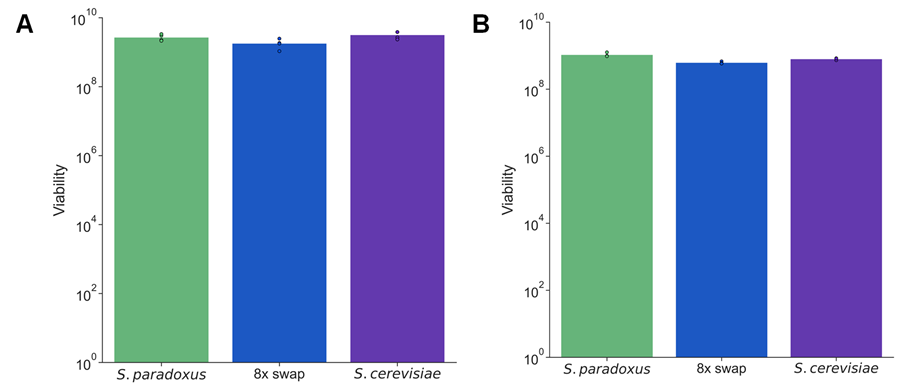

Supplement: S5 Fig — Data and symbols are as in Fig 3 of the main text except that liquid incubations were at 28°C. In no case was the respective measurement for a given strain significantly different from the analogous quantity for S. paradoxus at two-sided Wilcoxon p < 0.05. (TIF) [file pgen.1009793.s008.tif]

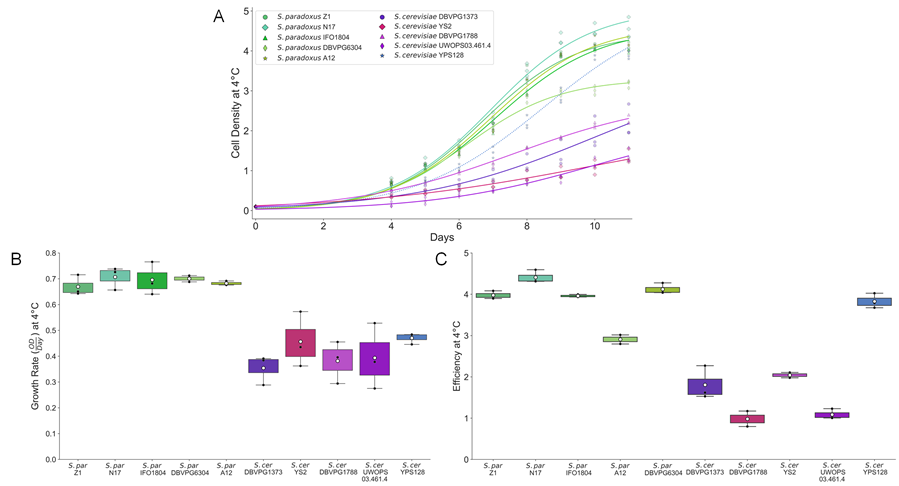

Supplement: S6 Fig — (A) Each trace reports a timecourse of growth at 4°C of the wild-type of the indicated strain. For a given strain, points on a given day report biological replicates; lines report the average fit from a logistic regression across replicates (S2 Table). YPS128, a North American S. cerevisiae known to have recently acquired freeze-thaw resistance as a derived character distinct from the ancestral program [64], is shown in faint blue. (B) The y-axis reports growth rate, in units of cell density (optical density, OD) per day, from the average logistic fit of the timecourse in (A) for the indicated strain. (C) The y-axis reports, for day 10 of the timecourse in (A) for the indicated strain, growth efficiency, the cell density after a 10-day incubation at 4°C as a difference from the starting density. In (B) and (C), Black points report individual biological replicates. White dots report means. Boxes span the interquartile range; whiskers are 1.5 times the interquartile range, and do not report outliers. Comparisons of growth rate and efficiency at 4°C between S. paradoxus strains and S. cerevisiae strains yielded Wilcoxon p ≤ 0.00003 and p ≤ 0.002, respectively. (TIF) [file pgen.1009793.s009.tif]

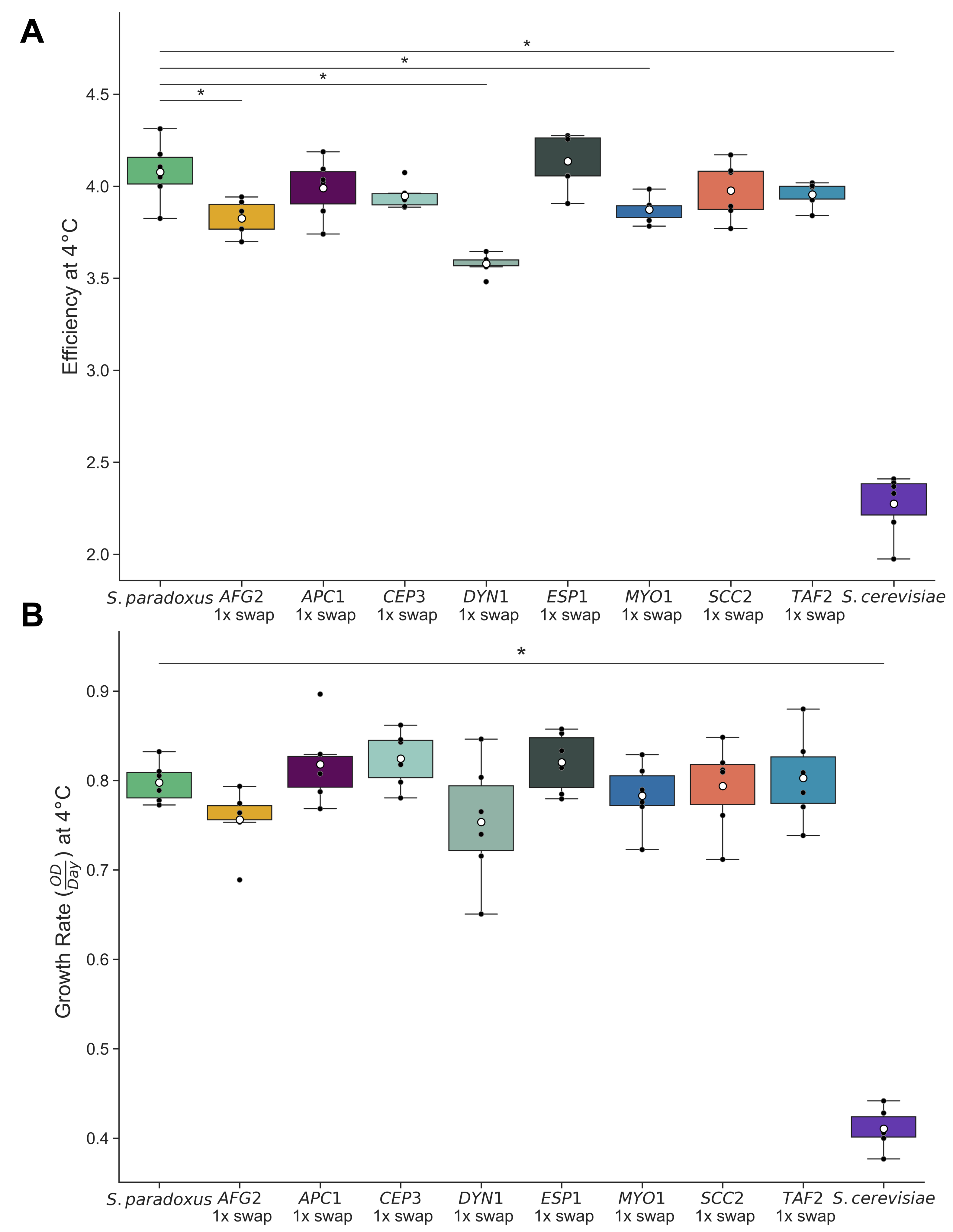

Supplement: S7 Fig — (A) Data and symbols are as in S3 Fig except that the y-axis reports growth efficiency after a 10-day incubation at 4°C. (B) Data are as in (A) except that the y-axis reports growth rate, in units of cell density (optical density, OD) per day, from the average logistic fit of the timecourse for the indicated strain (S2 Table). *, corrected one-sided Wilcoxon p ≤ 0.05. (TIF) [file pgen.1009793.s010.tif]

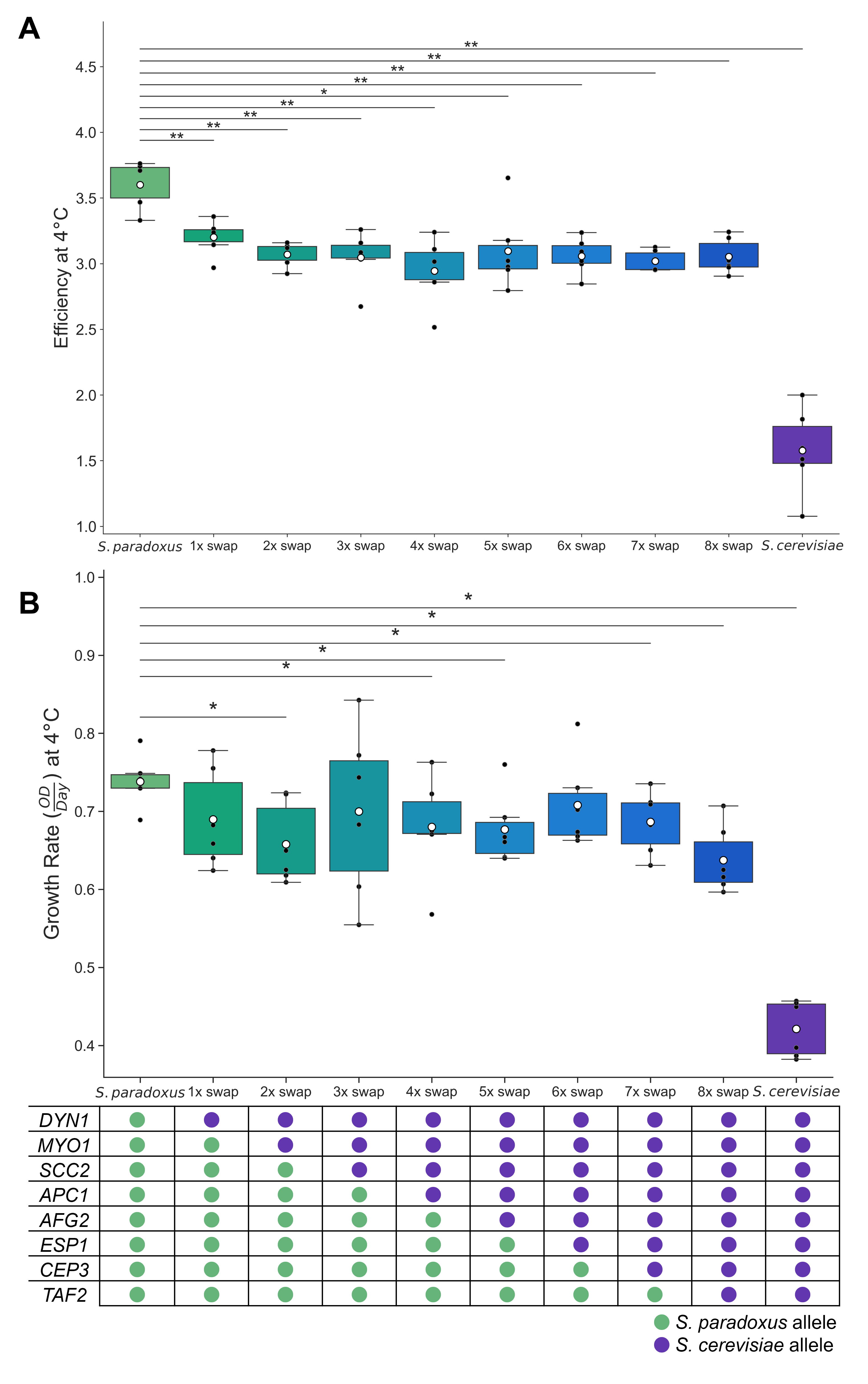

Supplement: S8 Fig — (A) Data and symbols are as in the main panel of Fig 1 except that the y-axis reports growth efficiency after a 10-day incubation at 4°C. (B) Data and symbols are as in (A) except that the y-axis reports growth rate, in units of cell density (optical density, OD) per day, from the average logistic fit of the timecourse for the indicated strain (S2 Table). * and **, corrected one-sided Wilcoxon p ≤ 0.05 and 0.01 respectively. (TIF) [file pgen.1009793.s011.tif]

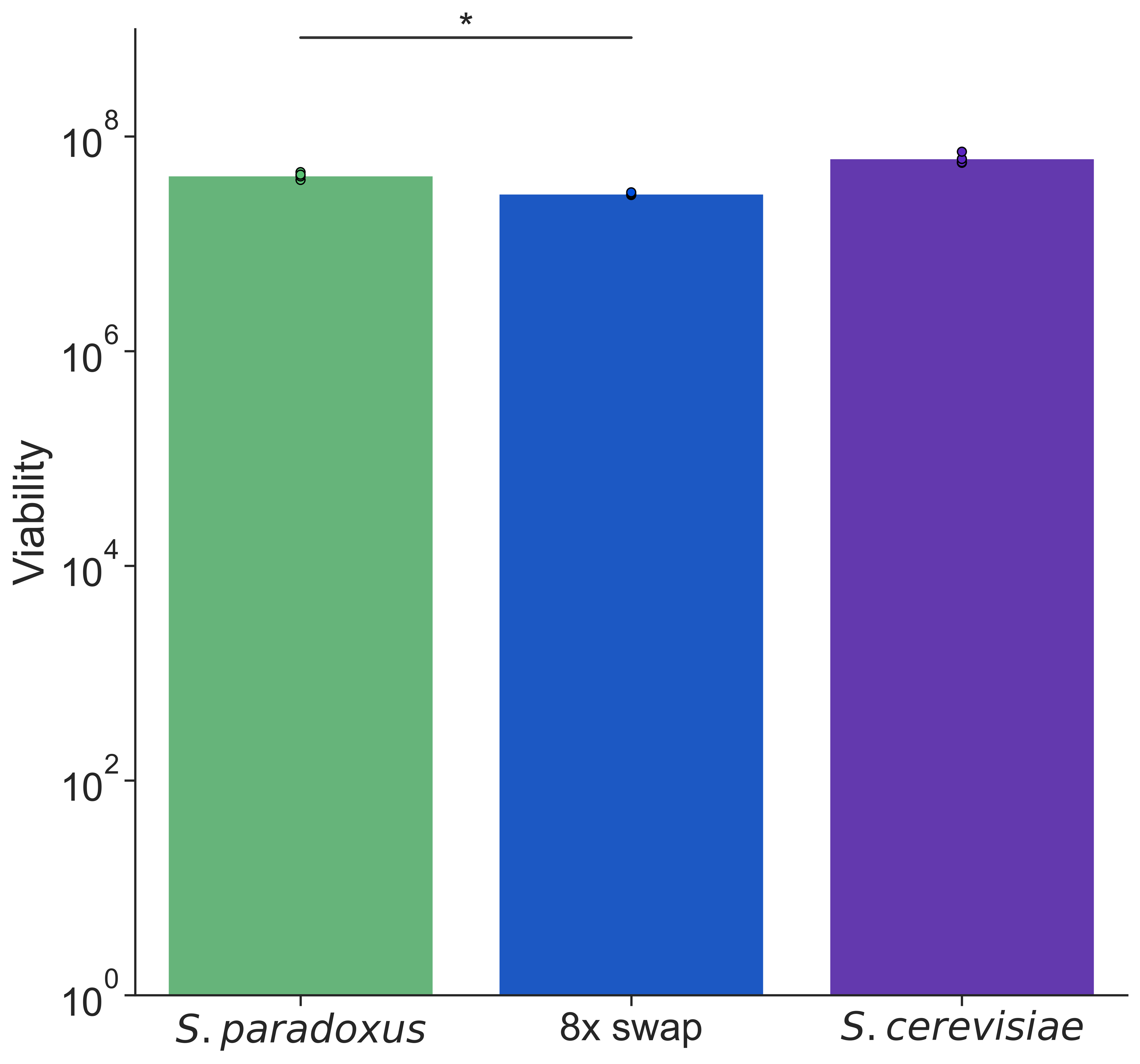

Supplement: S9 Fig — Data and symbols are as in Fig 3A of the main text, except that liquid incubations were at 4°C, and measurements were taken at day 8 of the growth timecourse. *, One-sided Wilcoxon p ≤ 0.05. (TIF) [file pgen.1009793.s012.tif]
